# Hybrid Steel/NSM/GFRP System versus GFRP Wrapping for Upgrading RC Wall-like Columns

**DOI:** 10.3390/polym15081886

**Published:** 2023-04-14

**Authors:** Hussein Elsanadedy, Husain Abbas, Nadeem Siddiqui, Tarek Almusallam, Yousef Al-Salloum

**Affiliations:** Chair of Research and Studies in Strengthening and Rehabilitation of Structures, Department of Civil Engineering, King Saud University, Riyadh 11421, Saudi Arabia

**Keywords:** RC wall-like columns, axial upgrading, GFRP wrapping, NSM bars, experimental study, FE analysis

## Abstract

Reinforced concrete (RC) wall-like columns are commonly employed in structures in Saudi Arabia. These columns are preferred by architects owing to their minimum projection in the usable space. However, they often need strengthening due to several reasons, such as the addition of more stories and increasing the live load as a result of changing the usage of the building. This research aimed to obtain the best scheme for the axial strengthening of RC wall-like columns. The challenge in this research is to develop strengthening schemes for RC wall-like columns, which are favored by architects. Accordingly, these schemes were designed so that the dimensions of the column cross-section are not increased. In this regard, six wall-like columns were experimentally examined in the event of axial compression with zero eccentricity. Two specimens were not retrofitted to be used as control columns, whereas four specimens were retrofitted with four schemes. The first scheme incorporated traditional glass fiber-reinforced polymer (GFRP) wrapping, while the second one utilized GFRP wrapping combined with steel plates. The last two schemes involved the addition of near-surface mounted (NSM) steel bars combined with GFRP wrapping and steel plates. The strengthened specimens were compared with regard to axial stiffness, maximum load, and dissipated energy. Besides column testing, two analytical approaches were suggested for computing the axial capacity of tested columns. Moreover, finite element (FE) analysis was performed for evaluating the axial load versus displacement response of tested columns. As an outcome of the study, the best strengthening scheme was proposed to be used by practicing engineers for axial upgrading of wall-like columns.

## 1. Introduction

RC wall-like columns have been frequently used in the multistory RC buildings of Saudi Arabia to save the space occupied by columns. These columns often need strengthening due to several reasons, such as the addition of more stories and increasing the live load as a result of changing the usage of the building. The traditional schemes for upgrading RC columns were the installation of RC jackets [1,2] and steel jackets [3,4,5,6,7] around the original column. However, these traditional techniques are time-consuming and difficult in their installation. Therefore, fiber-reinforced polymer (FRP) composites have been used recently in upgrading RC wall-like columns owing to their advantages, such as quick and easy installation, and almost negligible change in the column dimensions.

Most of the research conducted on concentrically loaded FRP-strengthened RC rectangular columns has dealt with sections having depth-to-width ratios varying from 1 to 2, and only a few studies [8,9,10,11,12,13,14,15,16] focused on FRP-upgraded wall-like columns. Tan [8] experimentally examined the peak axial load enhancement of FRP-upgraded wall-like columns with sections of depth/width ratio of 3.65. The peak load of tested columns was compared with existing analytical models [17,18]. Hosny et al. [9] examined the experimental response of FRP-upgraded wall-like columns with sections having a depth-to-width ratio of 3. Peak recorded FRP strains were much lower than the rupture values. Tanwongsval et al. [10] experimentally studied the performance of concentrically loaded unstrengthened and strengthened wall-like columns with sections having the same depth-to-width ratio as used in [8]. Two different techniques were used for column upgrading, which included traditional GFRP wrapping around the rectangular column section and GFRP wrapping after the section had been modified to a more rounded one. A better performance was observed for columns upgraded with the second scheme owing to improved concrete confinement. Maalej et al. [11] experimentally examined the impact of FRP retrofitting on the peak load enhancement of wall-like columns with sections having a depth/width ratio of 3.65. Besides the experimental program, an analytical model—suggested previously in [19,20]—was employed for computing the peak load of tested specimens.

Prota et al. [12] investigated via an experimental campaign the effect of wrapping GFRP sheets to increase the axial resistance of wall-like columns with sections having large depth-to-width ratios. It was concluded that GFRP wrapping could improve both the ductility and strength of columns. The failure of GFRP-strengthened columns depended on the section shape, and it occurred at horizontal GFRP strains much lower than the rupture values. De Luca et al. [13] studied GFRP-strengthened wall-like columns by testing three specimens (one control and two strengthened). In the strengthened specimens, two different GFRP confinement ratios were utilized. It was found that GFRP confinement did not potentially increase the ultimate load; however, it considerably enhanced the ultimate concrete strain.

Alsayed et al. [14] examined FRP-upgraded RC wall-like columns loaded under axial compression with zero eccentricity. The rectangular section was changed to an elliptical one via cementitious mortar, and it was thereafter wrapped by CFRP sheets. Nonlinear 3D FE analysis was also conducted for evaluating the load versus displacement response of tested specimens. CFRP wrapping increased both the ductility and strength of columns. Abbas et al. [15] further extended this study by considering some more schemes for the strengthening of wall-like columns. Triantafillou et al. [16] investigated the experimental response of FRP-upgraded wall-like columns. Forty-five columns with a section having depth-to-width ratios of 3 and 4 were tested under concentric compression. Different strengthening techniques were studied. They included unanchored and anchored CFRP confinement with or without section modification. The study concluded that the CFRP confinement effectiveness was almost doubled by the properly distributed anchors.

Based on the above-mentioned research, there are limited studies concerning the behavior of upgraded wall-like columns under axial compression. In this research, innovative hybrid steel/NSM/GFRP systems were suggested to upgrade RC wall-like columns loaded under axial compression with zero eccentricity. Compression tests were conducted on six specimens, of which two columns were not retrofitted and four specimens were retrofitted with different schemes. The schemes incorporated wrapping GFRP sheets around the column versus hybrid steel/NSM/GFRP systems. The strengthened specimens were compared with regard to axial stiffness, maximum load, and dissipated energy. Besides the testing campaign, two analytical approaches were suggested for computing the ultimate load of tested columns. Moreover, nonlinear FE analysis was performed for evaluating the behavior of tested columns. As an outcome of the study, the best strengthening scheme was proposed to be used by practicing engineers for axial upgrading of wall-like columns.

## 2. Experimental Study

The experimental study comprised axial compression tests conducted on wall-like columns. The investigated parameter in the experimental program was the retrofitting technique. Four strengthening schemes were experimentally studied.

### 2.1. Test Specimens

**Figure 1:**
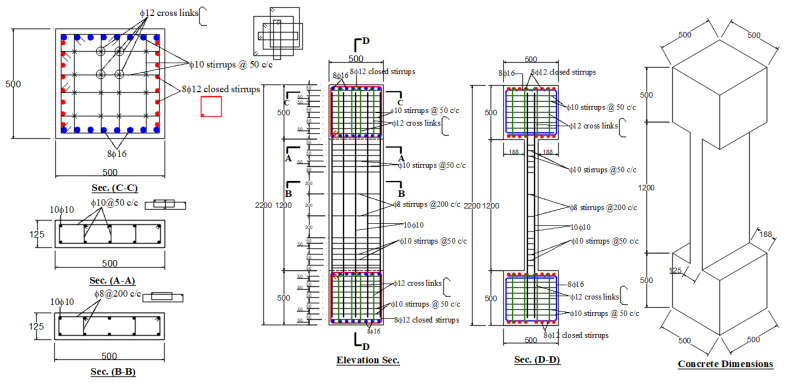
Details of control columns C1 and C2 (units of dimensions = mm).

A prototype column was chosen in the ground story of an existing office building with seven stories. The column was then half-scaled to be used as a control test specimen. As seen in Table 1, the test matrix comprises six columns with sections having a depth/width ratio of 4. It included two un-retrofitted columns [21] that were utilized as control specimens (C1 and C2). The other four columns (S1, S2, S3, and S4) were retrofitted with schemes 1, 2, 3, and 4, respectively. Figure 1 presents the details of control specimens (C1 and C2). Dimensions of the columns were 125 mm (width) × 500 mm (depth) × 1200 mm (height), and the vertical steel reinforcement was 10 Φ 10 mm. In the central 600 mm part of the height, transverse ties of Φ8 mm at a spacing of 200 mm were provided; in the end parts, transverse ties of Φ10 mm at a spacing of 50 mm were used. As illustrated in Figure 1, the ends of the specimen have RC boxes of dimensions 500 × 500 × 500 mm to minimize the stress concentration. The end boxes were heavily reinforced to remain uncracked throughout the test.

**Table 1:** Testing matrix.

Specimen ID	Upgrading Scheme	No. of Specimens
C1, C2	Control (unstrengthened) specimen (see Figure 1)	2
S1	GFRP wrapping (see Figure 2)	1
S2	GFRP wrapping + bolted steel plates (see Figure 3)	1
S3	GFRP wrapping + bolted steel plates + connected NSM steel bars (see Figure 4)	1
S4	GFRP wrapping + bolted steel plates + disconnected NSM steel bars (see Figure 5)	1
	Total No. of columns =	6

**Figure 2:**
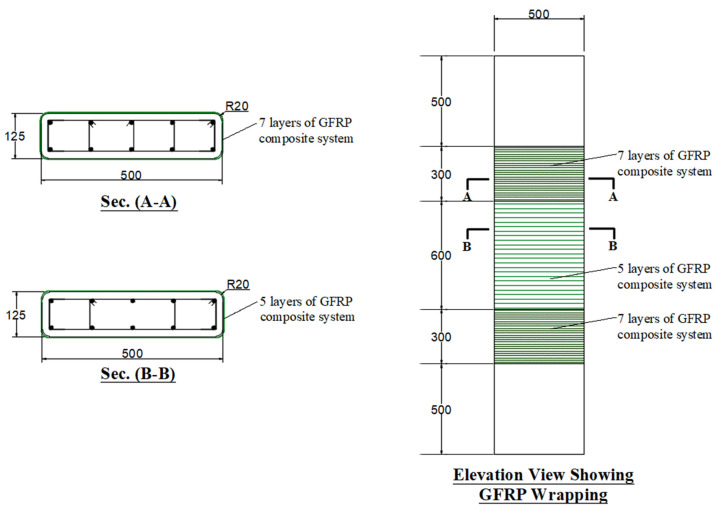
Details of strengthened column S1 (units of dimensions = mm).

In specimen S1, the first strengthening scheme (scheme 1) used the traditional GFRP wrapping. Details of specimen S1 are given in Figure 2. Five GFRP layers were employed in the central 600 mm length; nevertheless, in the top and bottom 300 mm length of the specimen, two extra GFRP layers were added for forcing failure to be in the central part of the specimen.

**Figure 3:**
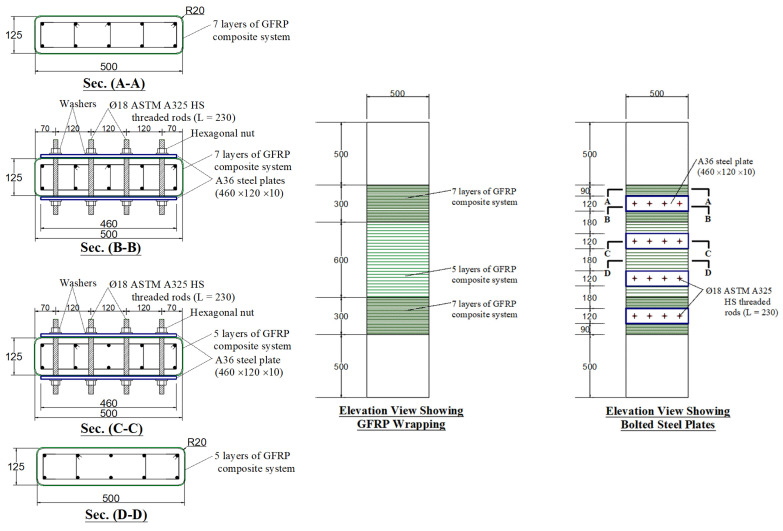
Details of strengthened column S2 (units of dimensions = mm).

In specimen S2, the second scheme of strengthening (scheme 2) used the externally bonded GFRP composite system of S1 combined with bolted steel plates. For each of the larger dimensions of the section, four steel plates of 460 × 120 mm size and 10 mm thickness were added at a vertical spacing of 300 mm (measured on centers). The steel plates provided on the two opposite faces were bolted together using high-strength steel rods (diameter = 18 mm). The steel plates were used for enhancing the confinement added by the GFRP wrapping. Figure 3 shows the details of this strengthening scheme.

**Figure 4:**
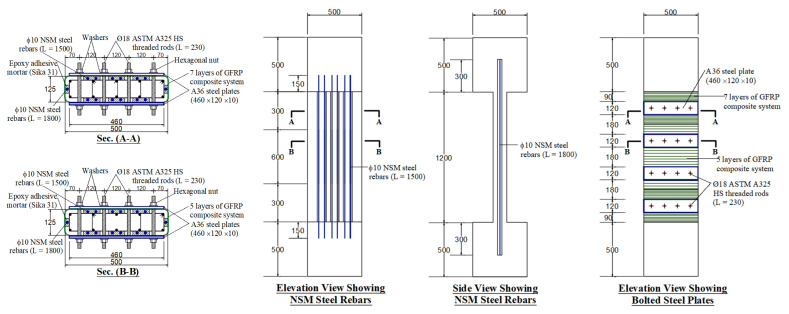
Details of strengthened column S3 (units of dimensions = mm).

In specimen S3, the third upgrading scheme (scheme 3) was a hybrid steel/NSM/GFRP system. Details of scheme 3 in specimen S3 are shown in Figure 4. It is the same as scheme 2 of specimen S2 with the addition of 14 Φ 10 mm vertical NSM steel bars, which increased the longitudinal steel ratio of the column from 1.26% to 3.0%. In this scheme, the externally bonded GFRP layers, along with the steel plates, were used to support the NSM bars laterally and hence delay their buckling until higher levels of axial stresses. As seen in Figure 4, the NSM bars in scheme 3 were continuously anchored with the end RC boxes with compression development length.

**Figure 5:**
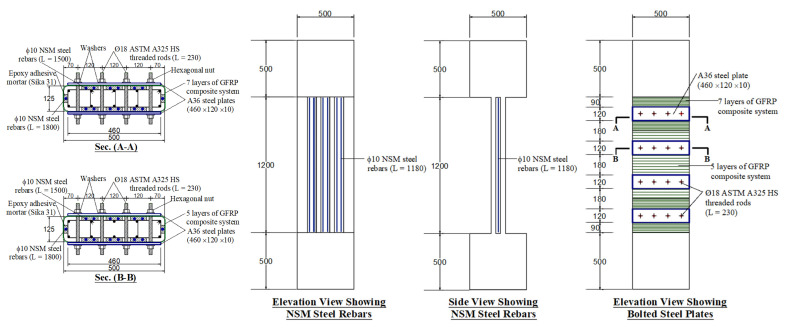
Details of strengthened column S4 (units of dimensions = mm).

Figure 5 presents details of strengthening scheme 4 in specimen S4. As illustrated in Figure 5, scheme 4 is identical to scheme 3 in specimen S3 (hybrid steel/NSM/GFRP system), but the NSM bars were detached from the end RC boxes. A gap of 10 mm width was left at the bar/RC bulb interface, as illustrated in Figure 5.

It should be noted that the investigated strengthening schemes are very economical owing to the use of locally available materials. The materials used in the studied schemes are GFRP composites, A36 steel plates, high-strength threaded rods, and steel rebars. Among the FRP composite materials used for structural applications, GFRP composites are the cheapest, and they are locally available. In addition, A36 steel plates, high-strength threaded rods, and steel rebars are locally manufactured, and hence they are available in the local market.

### 2.2. Material Properties

The mechanical properties of constituent materials are listed in Table 2. For the fabrication of columns, concrete with a target strength of 25 MPa was employed. Standard cylindrical concrete specimens (150 mm (diameter) × 300 mm (height)) were cast from the mix and then tested (as per [22]) to measure the compressive strength after 28 days and on the testing day of columns.

For high-strength rods and different diameters of steel bars, standard coupons were tested under uniaxial tension as per [23], and Table 2 presents the average mechanical properties. For GFRP composite sheets, standard coupons were tested under uniaxial tension as per [24], and Table 2 shows the average mechanical properties. As per [25], the longitudinal tensile strength seen in Table 2 for GFRP sheets was calculated as 0.55 times the average tensile strength of the flat coupons. For steel plates, standard coupons were tested as per [26], and the average mechanical properties are given in Table 2. For the epoxy-based mortar used with NSM bars, the mechanical properties given in Table 2 are provided by the datasheet of the manufacturer.

### 2.3. Preparation of Column Specimens

**Figure 6:**
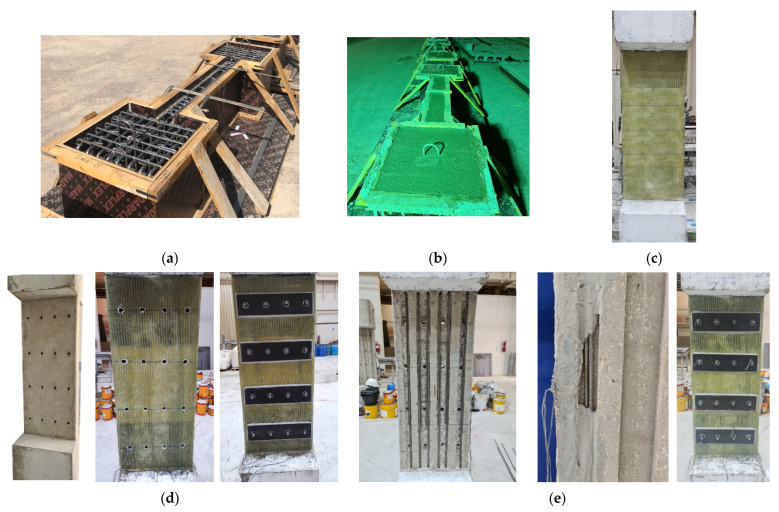
Preparation of columns: (**a**) reinforcement cage in the formwork before concrete casting; (**b**) column specimens after concrete casting; (**c**) strengthened specimen S1 after GFRP wrapping; (**d**) strengthening of specimen S2 using GFRP sheets combined with bolted steel plates; (**e**) strengthening of specimen S3 using GFRP sheets combined with bolted steel plates and connected NSM bars.

The steps involved in the preparation of columns are shown in Figure 6. The rebar cages in the formwork before concrete casting and the specimens after casting are presented in Figure 6a and Figure 6b, respectively. It should also be outlined that the corners in all upgraded columns were curved (radius = 20 mm) to minimize the concentration of stresses in the GFRP sheets at those locations (see Figure 2, Figure 3, Figure 4 and Figure 5).

For specimen S1, the surface was prepared via the standard procedure for GFRP wrapping [25]. The GFRP sheets were bonded onto the column via the standard wet-layup procedure [25]. Figure 6c shows the strengthened specimen S1 after GFRP wrapping.

Similar to scheme 1, the column surface of specimen S2 was prepared for GFRP wrapping. At the locations of threaded rods, holes (diameter = 20 mm) were drilled in the long sides of the section, as seen in Figure 6d. Similar to scheme 1, GFRP wrapping was conducted using the standard wet-layup procedure. After curing the GFRP sheets, holes were driven in the GFRP layers at the same locations on the concrete surface. Threaded rods of 18 mm diameter were inserted into the drilled holes, and they were bonded with concrete via an epoxy-based mortar. After curing the mortar, four steel strips of 460 × 120 × 10 mm size were adhesively bonded to the wider column face using epoxy-based mortar. Figure 6d shows the preparation steps of scheme 2.

In the last two strengthening schemes (specimens S3 and S4), steel NSM bars were added in pre-constructed grooves (see Figure 6e). The steel bars were bonded to the grooves using an adhesive mortar, as seen in Figure 6e. Upon mortar curing, the columns were strengthened following the same procedure used in specimen S2. In scheme 3, the NSM bars were anchored to the end bases; however, in specimen S4, the NSM bars were disconnected from the end bulbs (see Figure 5).

### 2.4. Sensor Layout and Test Setup

**Figure 7:**
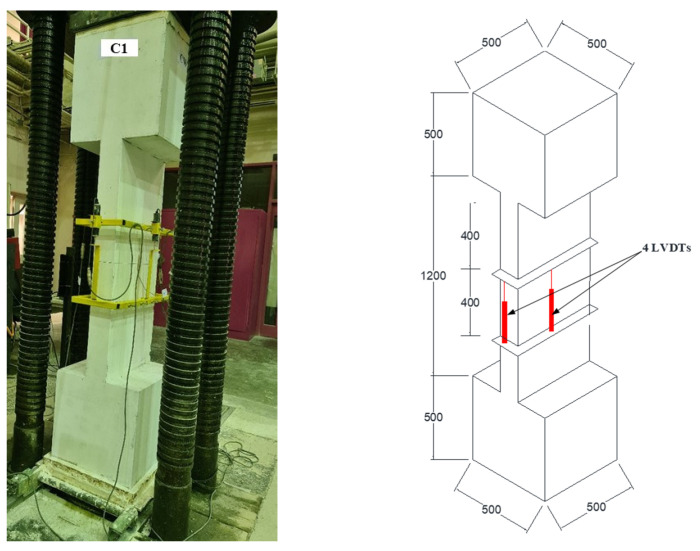
Test setup and instrumentation layout.

The layout of sensors and test setup for column specimens are presented in Figure 7. A compression machine (capacity = 10,000 kN) was employed for applying concentric axial compression on the columns. As seen in Figure 7, four LVDTs were connected to the middle portion for measuring the axial displacement. Electric resistance strain gages were also attached to the concrete surface, steel bars, steel plates, and GFRP sheets to record their strains during the test. The experimental results were recorded with the help of a data acquisition system.

## 3. Discussion of Test Results

**Figure 8:**
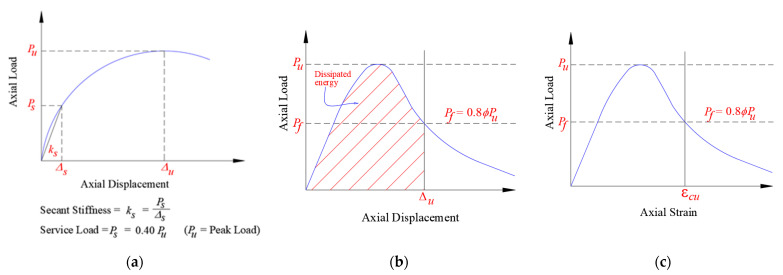
Definition of response parameters for column specimens [7,27,28]: (**a**) secant stiffness; (**b**) dissipated energy; (**c**) ultimate concrete strain.

The main test results with regard to load versus displacement behavior are shown in Table 3 for tested columns. These are the yield and peak loads; the axial displacements at service load, yield load, ultimate load, and ultimate state; the axial secant stiffness (computed at service load); and the energy dissipated. As clarified in Figure 8a, the service load (*P_s_*) was taken as 0.4 times the maximum load (*P_u_*) [7,27,28]. The axial secant stiffness (*k_s_*) was calculated as the ratio between the service load (*P_s_*) and the corresponding displacement (*Δ_s_*), as seen in Figure 8a. The energy dissipated was calculated at the ultimate state, as shown in Figure 8b. In this study, the ultimate state was assumed as that associated with the crushing of concrete (see Figure 8b,c). It is taken as that related to a failure load equaling the peak design load as per the codes [29,30].

The experimental results of the stress versus strain response of column specimens are listed in Table 4. These results are the maximum average and actual concrete strengths, the axial concrete strains at maximum stress and ultimate state, the strains in steel rebars at maximum load, and the maximum horizontal strains in GFRP sheets and steel plates. The maximum average $f_{c-avg}^{\prime }$ and actual concrete $f_{c-act}^{\prime }$ strengths were computed from the following equations:

(1) $$f_{c-avg}^{\prime }=\frac{P_{u}}{A_{g}}$$

(2) $$f_{c-act}^{\prime }=\frac{P_{u}-A_{st}f_{y-st}-A_{NSM}f_{y-NSM}}{A_{g}-A_{st}-A_{NSM}}$$

where *P_u_* is the peak load; *A_g_* is the whole area of the column section; *A_st_* and *A_NSM_* are the areas of original and NSM bars, respectively; and *f_y-st_* and *f_y-NSM_* are the yield strengths of original and NSM bars, respectively.

**Figure 9:**
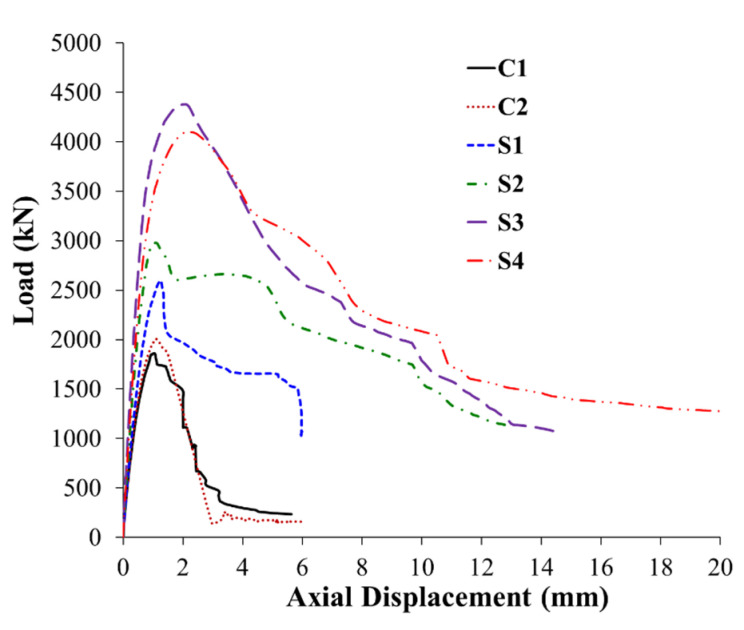
Load versus displacement curves for column specimens based on test results.

**Figure 10:**
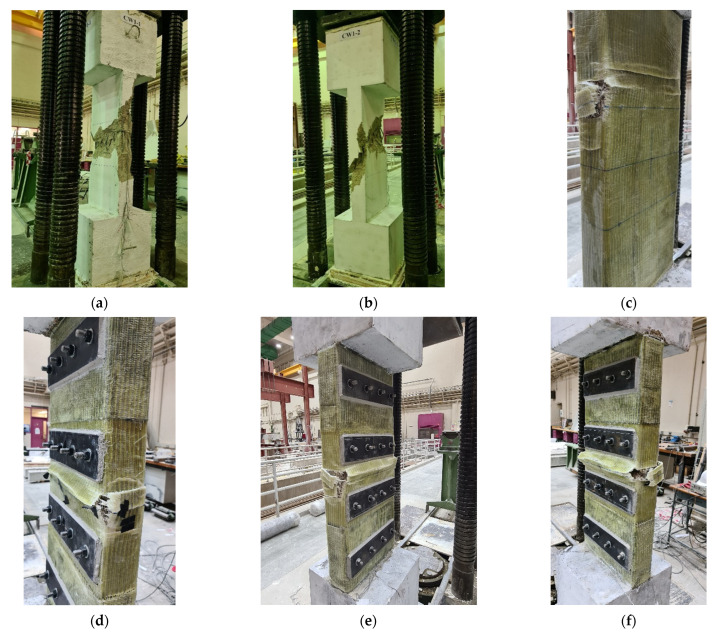
Observed failure modes of columns: (**a**) C1; (**b**) C2; (**c**) S1; (**d**) S2; (**e**) S3; (**f**) S4.

The experimental load–displacement plots for the six tested columns are displayed in Figure 9. The observed failure modes of tested columns are presented in Figure 10. In the next subsections, the experimental results of the control and strengthened columns are discussed.

### 3.1. Control Specimens

As presented in Table 3 and Figure 9, the ultimate loads of the two control columns, C1 and C2, were 1862 and 2006 kN, respectively [21]. The axial resistance of the control columns continued to decrease after reaching the peak load until it dropped down to very small values. Figure 10a,b displays the failure modes for columns C1 and C2, respectively. Both columns had a common brittle failure that started with spalling of the concrete cover at high axial strains. This was succeeded by buckling of vertical bars and then crushing of concrete in the middle part of the column, as seen in Figure 10a,b.

### 3.2. Strengthened Specimens

#### 3.2.1. Column S1

This column was retrofitted with scheme 1 (GFRP wrapping). As presented in Table 3 and Figure 9, the ultimate load of the column was 2596 kN, which is 34% more than the average maximum load of the control columns C1 and C2. It is also depicted that GFRP wrapping significantly enhanced the ultimate displacement, as it was about 76% higher than the average value of the control columns. Figure 10c shows the observed failure mode of strengthened column S1. The column failure originated from the bulging of its section, which caused an outward push on the GFRP sheets in the central 600 mm length, where the tie spacing was 200 mm. Upon reaching the ultimate load, there was a sudden small drop in the axial capacity. The increase in the bulging of the column section and the consequent buckling of the vertical bars, especially those on the short side of the column, resulted in the rupture of GFRP sheets on the smaller side of the section and caused column failure at a very high axial strain (see Figure 10c).

#### 3.2.2. Column S2

This column was strengthened using scheme 2, which employed five layers of externally bonded GFRP sheets in the middle part (same as employed in S1) together with bolted horizontal steel plates. The peak load achieved for this strengthening scheme was 2978 kN, which was 54% more than the control columns. This scheme was also better than the GFRP wrapping scheme adopted in scheme 1, as the ultimate load of the S2 column was 15% more than that of specimen S1. The ultimate load enhancement (over S1) was owing to the presence of horizontal steel plates, which improved the confinement efficiency of GFRP wrapping. It should be clarified that the addition of bolted steel plates in scheme 2 not only enhanced the ultimate load but also significantly improved the ultimate displacement. As noted from Table 3 and Figure 9, the ultimate displacement of column S2 is higher than that of the control specimens and strengthened column S1 by about 253% and 100%, respectively. Figure 10d depicts the failure mode of the retrofitted specimen S2. The failure originated from the bulging of the GFRP sheets between the steel plates at the central part, which caused a small drop in the axial capacity. The failure was ended by the rupture of the GFRP wrapping close to the column corner due to the buckling of bars on the smaller column side.

#### 3.2.3. Column S3

This specimen was strengthened by a hybrid system consisting of continuous NSM steel bars combined with both GFRP wrapping and bolted horizontal steel plates. As identified in Figure 9 and Table 3, the ultimate load of column S3 was 4379 kN, which is more than the average maximum load of control specimens C1 and C2 by about 126%. This scheme was also better than schemes 1 and 2, as the ultimate load of the S3 column was 69% and 47% more than that of specimens S1 and S2, respectively. The ultimate load improvement over S2 was owing to the contribution of NSM bars in both confining the concrete core and increasing the axial resistance. As depicted in Table 3 and Figure 9, the ultimate displacement of specimen S3 was considerably higher than control columns and strengthened specimen S1 by about 157% and 46%, respectively. However, it was less than that of specimen S2 by about 27%, owing to the buckling of NSM bars in specimen S3. In conclusion, scheme 3 is a superb scheme, and it is the most efficient in terms of upgrading the axial load resistance of the columns. Figure 10e displays the observed failure mode of column S3. The failure originated from the bulging of the GFRP sheets between the steel plates at the central part of the height, which caused a drop in the load. The final failure was caused by the rupture of the GFRP wrapping near the column corner, owing to the buckling of both the original and NSM bars on the smaller side of the column, as seen in Figure 10e.

#### 3.2.4. Column S4

This specimen was upgraded by the fourth scheme, which is identical to the third scheme, but the NSM bars were detached from the end bulbs. The ultimate load improvement for specimen S4 is 112% compared with the control columns C1 and C2. The ultimate load of column S4 was 4105 kN, which is more than that of specimens S1 and S2 by 58% and 38%, respectively. Nevertheless, it is less than the maximum load of column S3 by about 6%. As seen from Table 3 and Figure 9, the ultimate displacement of column S4 was considerably more than that control columns and strengthened specimen S1 by about 202% and 71%, respectively. However, it was less than that of specimen S2 by about 15% due to the buckling of NSM bars. Compared with specimen S3, the ultimate displacement of specimen S4 was higher by about 17%. Figure 10f shows the observed failure mode of specimen S4. The failure is almost identical to column S3, and it originated from the bulging of the GFRP sheets between the steel plates at the central part of the column height, which caused a drop in the load. The final failure was by the rupture of GFRP wrapping near the column corner owing to the buckling of both the original and NSM bars on the smaller side of the column, as seen in Figure 10f. As distinguished from the observations of the experiments, the detached NSM bars in column S4 entirely contributed to resisting the load until their buckling. Thus, the difference in peak load and ultimate displacement between specimens S3 and S4 could be attributed to the variation in material properties.

## 4. Analytical Study

The ultimate load of control and strengthened specimens was computed using two different approaches. The first approach employed the models of both the ACI 318 code [29] and the ACI 440.2R guidelines [25]; however, the second approach used the models of both Eurocode 2 [31] and the ACI 440.2R guidelines [25]. The calculation steps for the two analytical approaches are detailed as follows:

### 4.1. Approach 1

For control column C, the ACI 318 code [29] was utilized to compute the peak load (*P_u_*) from

(3) $$P_{u}=0.85f_{c}^{\prime }(A_{g}-A_{st})+f_{y-st}A_{st}$$

where the definition of the used symbols has been provided earlier in Section 3.

For strengthened column S1, the initial step is to compute the confined concrete strength ($f_{cc}^{\prime }$) via the following formulas of the ACI 440.2R guidelines [25].

(4) $$f_{cc}^{\prime }=f_{c}^{\prime }+3.3\Psi_{f}k_{a}f_{𝓁}$$

where Ψ*_f_* = 0.95 and *k_a_* is an FRP confinement efficiency factor calculated from

(5) $$k_{a}=\frac{A_{e}}{A_{c}}{\left(\frac{B}{H}\right)}^{2}$$

(6) $$\frac{A_{e}}{A_{c}}=\frac{1-\frac{\left[\left(\frac{B}{H}\right){\left(H-2r_{c}\right)}^{2}+\left(\frac{H}{B}\right){\left(B-2r_{c}\right)}^{2}\right]}{3A_{g}}-\rho_{g}}{1-\rho_{g}}$$

where *H* and *B* are the depth and width of the column, respectively; *r_c_* is the radius of the corner; and *ρ_g_* is the ratio of vertical steel bars = *A_st_/BH*. In Equation (4), the symbol $f_{𝓁}$ is the peak confinement stress given by the GFRP jacket, and it is calculated from

(7) $$f_{𝓁}=\frac{2E_{f}t_{f}\varepsilon_{fe}}{D_{e}}$$

where *E_f_* is the modulus of elasticity of GFRP sheets; *t_f_* is the total thickness of GFRP layers; *ε_fe_* is the effective strain of GFRP sheets (assumed as 55% of the rupture strain); and *D_e_* is the diagonal of the rectangular section, computed from

(8) $$D_{e}=\sqrt{B^{2}+H^{2}}$$

The peak axial load of upgraded column S1 was then calculated from Equation (3) after replacing $f_{c}^{\prime }$ with $f_{cc}^{\prime }$.

For upgraded column S2, the bolted steel plates were assumed to be smeared into an equivalent GFRP jacket with its equivalent thickness given by

(9) $$t_{eq}=\frac{t_{p}E_{s}b_{p}}{E_{f}S_{p}}$$

where *t_p_* is the thickness of steel plates (=10 mm); *E_s_* is Young’s modulus of steel plates (=2 × 10^5^ MPa); *b_p_* is the width of steel plates (=120 mm); and *S_p_* is spacing of steel plates measured on centers (=300 mm). The peak confinement stress given by the GFRP jacket combined with steel plates was then computed from

(10) $$f_{𝓁}=\frac{2E_{f}\varepsilon_{fe}\left(t_{f}+t_{eq}\right)}{D_{e}}$$

Equations (4)–(6) were utilized to assess the confined concrete strength ($f_{cc}^{\prime }$), and the peak load of upgraded column S2 was computed from Equation (3) after replacing $f_{c}^{\prime }$ with $f_{cc}^{\prime }$.

For upgraded specimen S3, Equation (10) was utilized to calculate the peak confinement stress added by the GFRP jacket combined with bolted steel plates ($f_{𝓁}$). Thereafter, the confined concrete core strength ($f_{cc}^{\prime }$) was computed from Equation (4), and the confined mortar grout strength $f_{cc-grout}^{\prime }$ (used in the NSM grooves) was calculated from Equation (4) after replacing $f_{c}^{\prime }$ with the unconfined grout mortar strength $f_{c-grout}^{\prime }$ (=65 MPa). The ultimate axial load of strengthened column S3 was then assessed from

(11) $$P_{u}=0.85f_{cc}^{\prime }(A_{g}-A_{grout}-A_{st})+0.85f_{cc-grout}^{\prime }(A_{grout}-A_{NSM})+f_{y-st}A_{st}+f_{y-NSM}A_{NSM}$$

As demonstrated from the test results, the detached NSM bars in column S4 fully contributed to resisting the load until their buckling. Therefore, the analytically predicted ultimate loads of columns S3 and S4 are identical.

### 4.2. Approach 2

For control column C, Eurocode 2 [31] was employed to predict the peak load from

(12) $$P_{u}=\eta f_{c}^{\prime }(A_{g}-A_{st})+f_{y-st}A_{st}$$

where *η* is the equivalent rectangular stress block parameter, computed from

(13) $$\eta =\left\{\begin{array}{l} 1.0\;\mathrm{for}\;f_{c}^{\prime }\leq 50\;\mathrm{MPa}\\ \frac{250-f_{c}^{\prime }}{200}\;\mathrm{for}\;50\;<\;f_{c}^{\prime }\leq 90\;\mathrm{MPa} \end{array}\right.$$

For upgraded column S1, the peak load was predicted from Equation (12) after replacing $f_{c}^{\prime }$ with the confined concrete compressive strength $f_{cc}^{\prime }$, computed from

(14) $$f_{cc}^{\prime }=\left\{\begin{array}{l} f_{c}^{\prime }\left(1.0+5.0\frac{f_{𝓁}^{\prime }}{f_{c}^{\prime }}\right)\;\mathrm{for}\;f_{𝓁}^{\prime }\leq 0.05f_{c}^{\prime }\\ f_{c}^{\prime }\left(1.25+2.5\frac{f_{𝓁}^{\prime }}{f_{c}^{\prime }}\right)\;\mathrm{for}\;f_{𝓁}^{\prime }>0.05f_{c}^{\prime } \end{array}\right.$$

where $f_{𝓁}^{\prime }$ is the effective confinement stress owing to GFRP wrapping, estimated from

(15) $$f_{𝓁}^{\prime }=k_{a}f_{𝓁}$$

where *k_a_* is an efficiency factor calculated using Equations (5) and (6) and $f_{𝓁}$ is the peak confinement stress of the GFRP jacket calculated from Equation (7).

For strengthened column S2, the peak load was computed using the same procedure used in specimen S1, except that the peak confinement stress $f_{𝓁}$ was estimated using Equations (9) and (10).

For upgraded columns S3 and S4, Equations (9) and (10) were used to estimate the peak confinement stress $f_{𝓁}$ provided by the GFRP jacket combined with bolted steel plates. Then, the effective confinement stress $f_{𝓁}^{\prime }$ was estimated using Equation (15), and the confined concrete core strength ($f_{cc}^{\prime }$) was estimated from Equation (14). The compressive strength of confined grout mortar ($f_{cc-grout}^{\prime }$) in the NSM grooves was also predicted from Equation (14) after replacing $f_{c}^{\prime }$ with the unconfined grout mortar strength $f_{c-grout}^{\prime }$ (=65 MPa).

The maximum axial load of upgraded columns S3 and S4 was then calculated from

(16) $$P_{u}=\eta f_{cc}^{\prime }(A_{g}-A_{grout}-A_{st})+\eta f_{cc-grout}^{\prime }(A_{grout}-A_{NSM})+f_{y-st}A_{st}+f_{y-NSM}A_{NSM}$$

Table 5 presents the maximum load assessed by Approaches 1 and 2 for the six tested columns. Thus, the analytical loads were compared with the experimental values. It is clarified from Table 5 that Approach 1 assesses the ultimate load of control specimens C1 and C2 more precisely than Approach 2. In Approach 1, the ultimate load of C1 was overestimated by 4%, but the ultimate load of C2 was underestimated by 3%. However, in Approach 2, the maximum loads of control columns C1 and C2 were overestimated, respectively, by 16% and 9%. It is also noted from Table 5 that Approach 2 assesses the ultimate load of upgraded columns S1 to S4 more precisely than Approach 1. The prediction errors in Approach 1 varied from 20% to 35%; however, the prediction errors in Approach 2 ranged from 3% to 14%. Accordingly, a more precise prediction process that closely matches the experimental results of the tested columns is highly required. In this respect, nonlinear FE analysis was performed for evaluating the behavior of un-retrofitted and retrofitted columns.

## 5. FE Analysis

Nonlinear FE analysis was conducted for the numerical evaluation of the load–displacement response of tested columns. In this respect, the commercially available package LS-DYNA [32] was employed.

### 5.1. FE Mesh

**Figure 11:**
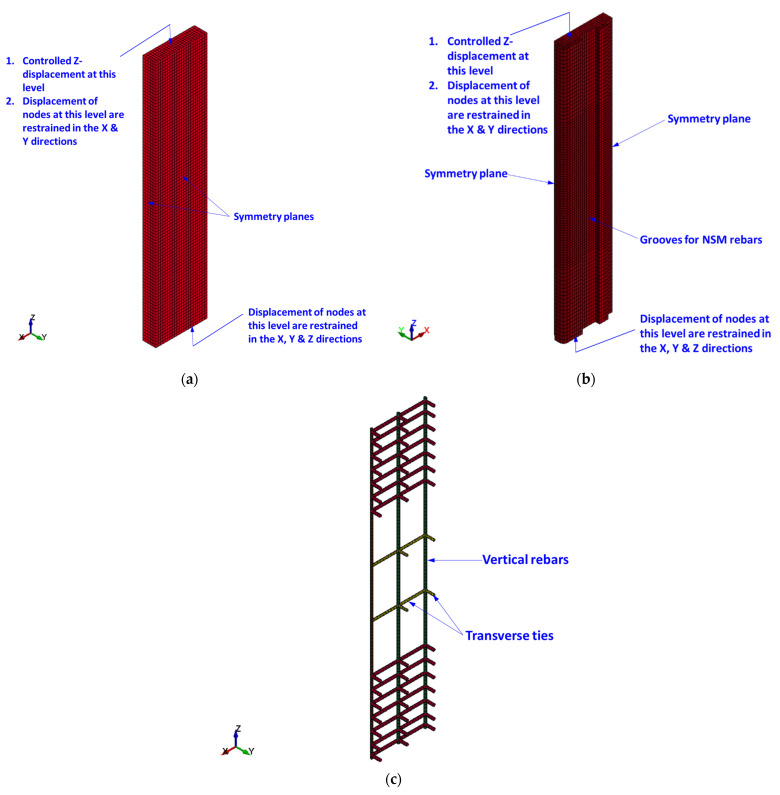
FE geometry for concrete volume and steel reinforcement of specimens: (**a**) concrete mesh for specimens C, S1, and S2; (**b**) concrete mesh for specimens S3 and S4; (**c**) mesh of reinforcement cage of all specimens.

**Figure 12:**
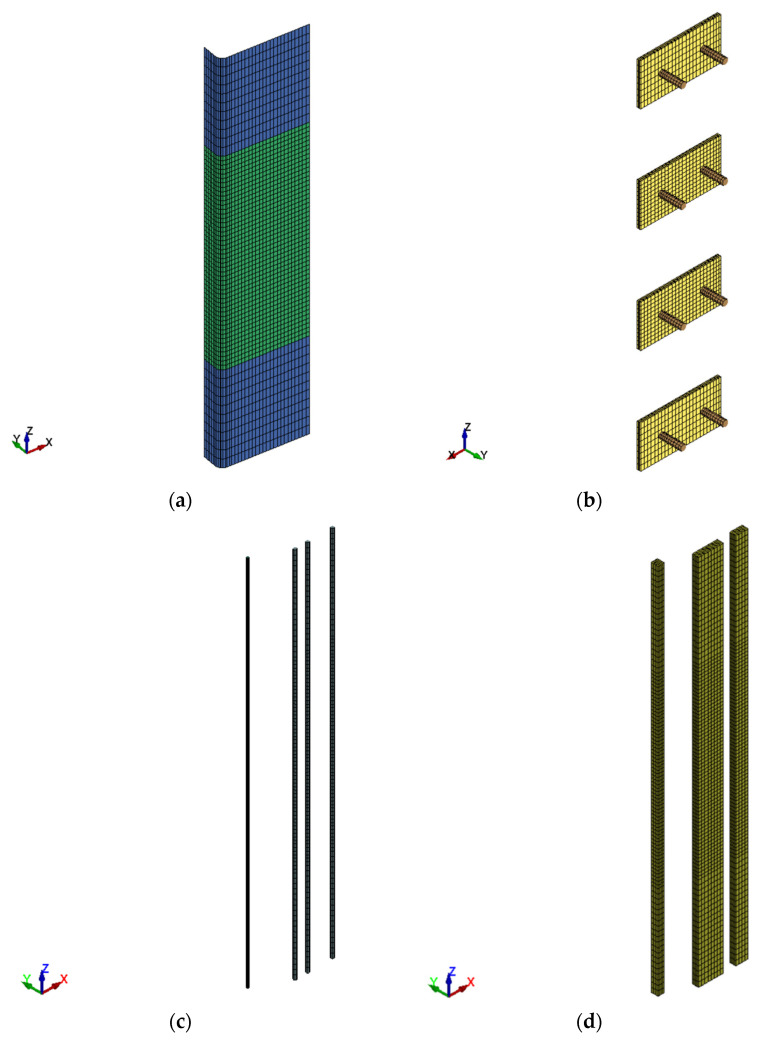
FE geometry for strengthening schemes: (**a**) GFRP sheets for upgraded columns; (**b**) steel plates with rods for specimens S2, S3, and S4; (**c**) NSM bars for specimens S3 and S4; (**d**) epoxy-based mortar for specimens S3 and S4.

The FE mesh for different column components is presented in Figure 11 and Figure 12. As the column specimens are symmetric about both XZ and YZ planes with regard to geometry and dimensions of constituent parts (concrete, steel reinforcement, and strengthening systems), properties of constituent materials, boundary conditions, and loading, only one-quarter of the column was modeled (see Figure 11 and Figure 12). This was done in order to considerably reduce the size of the model and hence reduce the computational time and save disk space. It is noted from Figure 11 that the RC end bases were not modeled as they had a linear elastic behavior during the experiments without signs of crushing and/or cracking. Brick elements were employed for modeling concrete, mortar, and steel plates. Nevertheless, beam and shell elements were, in turn, utilized for representing steel bars and GFRP wrapping. Full bond behavior was modeled at bar/concrete and GFRP sheet/concrete interfaces. A maximum element size of 25 mm was utilized, and a finer mesh was unnecessary based on a conducted convergence study.

### 5.2. Constitutive Models

Both the mortar and concrete volumes were modeled using the concrete damage model type 72R3, which was developed in [33,34,35]. This model uses three individual failure surfaces for the definition of the deviatoric strength [32]. For modeling steel plates, bars, and threaded rods, the type 24 plasticity model was utilized with a bilinear stress–strain curve [32]. For modeling GFRP sheets, the type 54–55 composite damage model was utilized with Change and Change failure parameters [36]. The key input parameters of the constitutive models are listed in Table 2.

### 5.3. Modeling of Supports and Loading Protocol

**Figure 13:**
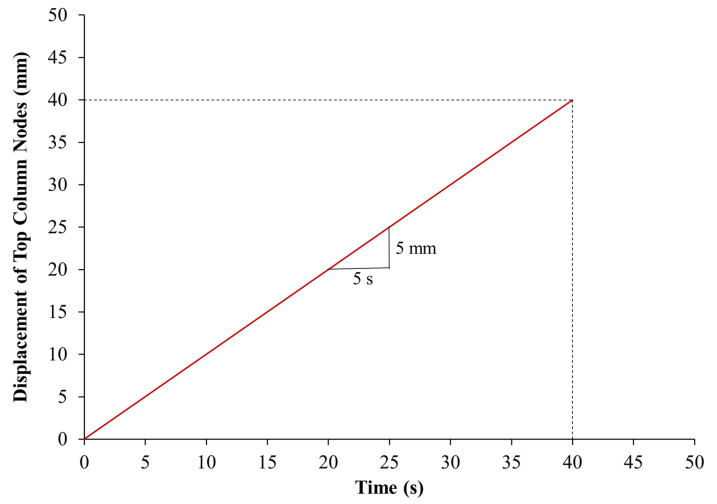
Displacement–time history for top column nodes.

In order to represent the support conditions of the experiments, the bottommost nodes of the column model were restrained against translation in all global directions; nevertheless, the topmost nodes were prevented from the X and Y translations only. For the one-quarter column model, symmetry boundary conditions were applied for the two symmetry planes, as seen in Figure 11. For modeling the loading protocol used in the experiments, the displacement–time curve seen in Figure 13 was applied for the topmost nodes of the column model.

## 6. Discussion of Numerical Results

**Figure 14:**
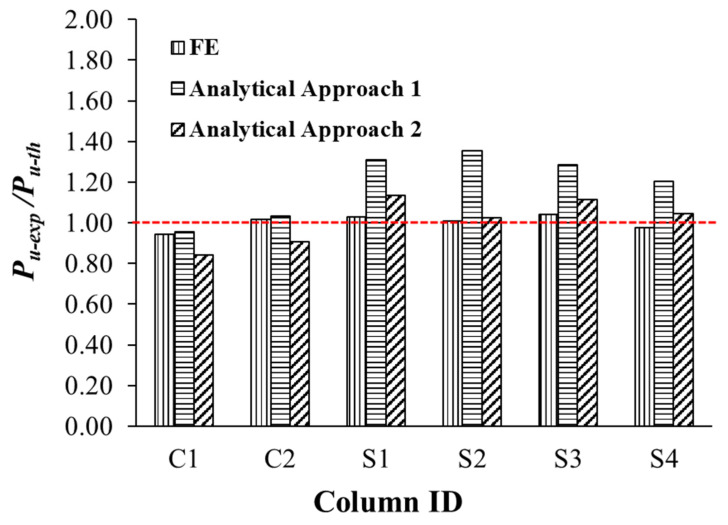
Tested-to-predicted ultimate load ratio for column specimens.

The comparisons between the numerical and test results with respect to the load–displacement behavior of the tested columns are summarized in Table 3. The comparative parameters are yield and maximum loads; axial displacements at service load, yield load, ultimate load, and ultimate state; axial stiffness; and dissipated energy at ultimate state. The experimental-to-predicted ratios of all response parameters are also given in Table 3. The mean values of the experimental-to-predicted ratios of all response parameters of the two control columns [21] are also presented in the table. For the numerical prediction of yield and ultimate loads, the errors were 3–11% and 1–6%, respectively. For the numerical prediction of axial displacements, the errors were 0–18%. Nevertheless, the errors in the numerical prediction of stiffness and dissipated energy were 4–16% and 2–15%, respectively. Figure 14 illustrates the tested-to-calculated ultimate load ratios ($P_{u-\exp}/P_{u-th}$) of the six tested columns for FE analysis and both analytical approaches 1 and 2. It is noted that the FE analysis estimates the ultimate load of all specimens more accurately than the proposed analytical approaches.

The comparisons between the numerical and test results with respect to the stress–strain behavior of the tested columns are given in Table 4. The comparative parameters are the maximum average and actual compressive strength of concrete (prediction errors were 1–6% and 1–7%, respectively), the axial concrete strains at maximum stress and ultimate state (errors were 0–8% and 4–18%, respectively), the strains in the original vertical bars at ultimate load (prediction errors were 4–11%), the strains in the NSM bars at maximum load (errors were 4–9%), and the maximum horizontal strains in GFRP sheets and steel plates (errors were 3–19% and 11–16%, respectively).

As noted from the FE results presented in Table 3 and Table 4, the FE results of upgraded columns S3 and S4 are equal. Therefore, it can be outlined that the detached NSM bars in column S4 entirely contributed to resisting the load until their buckling at the analysis end. Detailed discussions of the numerical results of column specimens are given below.

### 6.1. Modes of Failure

**Figure 15:**
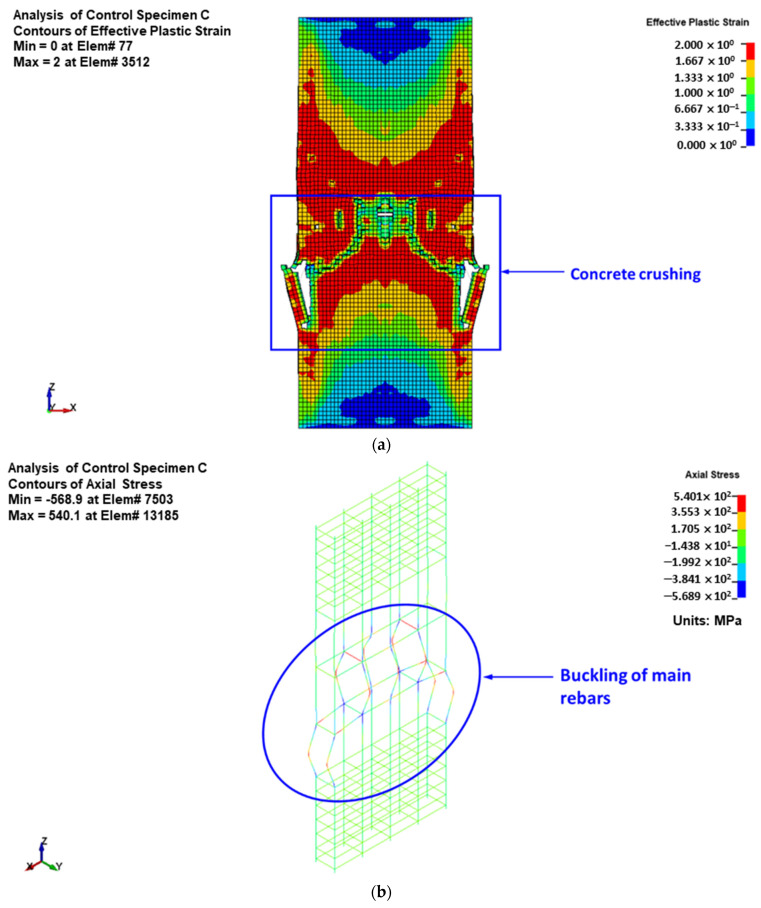
Numerically predicted failure mode for control column C: (**a**) concrete damage contours; (**b**) axial stress contours for reinforcement cage.

**Figure 16:**
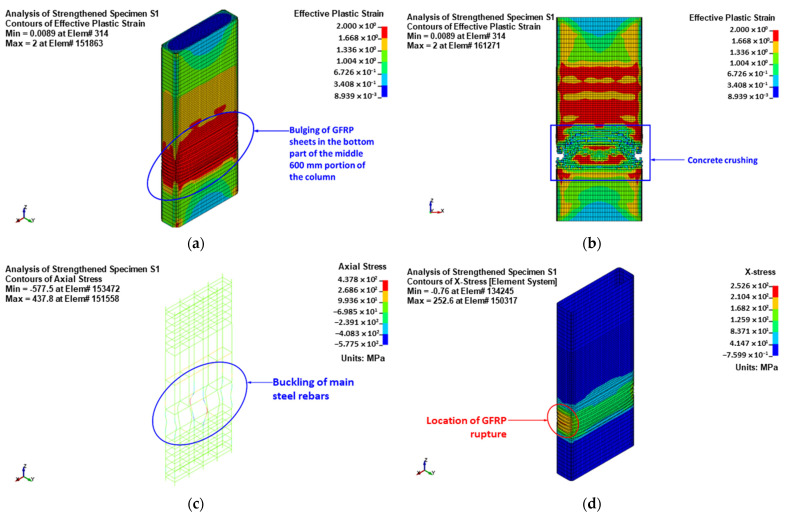
Numerically predicted failure mode for upgraded column S1: (**a**) bulging of GFRP sheets at maximum load; (**b**) concrete damage contours; (**c**) stresses in reinforcement cage; (**d**) stresses in the fiber direction for GFRP sheets.

**Figure 17:**
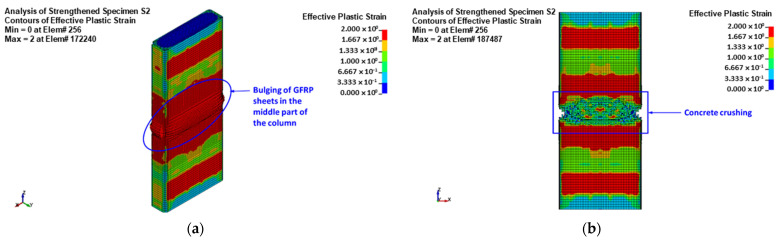

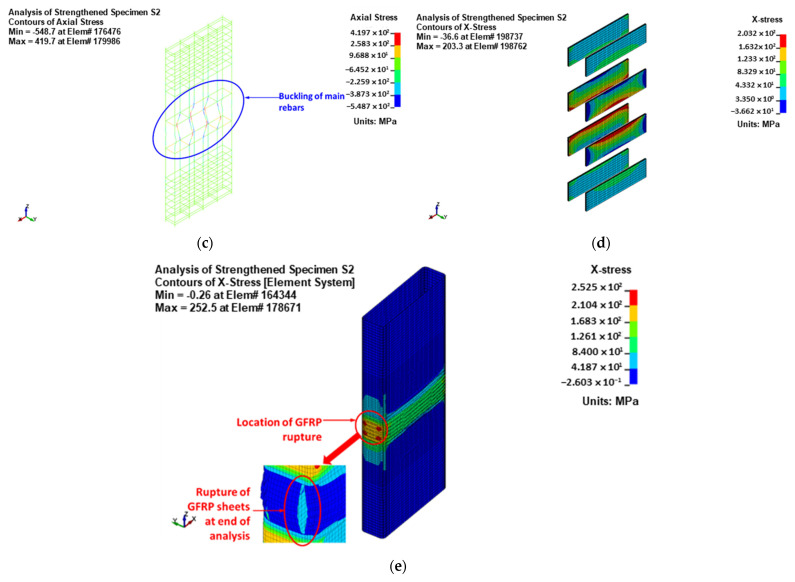
Numerically predicted failure mode for upgraded column S2: (**a**) bulging of GFRP sheets at maximum load; (**b**) concrete damage contours; (**c**) stresses in reinforcement cage; (**d**) stresses in steel plates; (**e**) stresses in the fiber direction for GFRP sheets.

**Figure 18:**
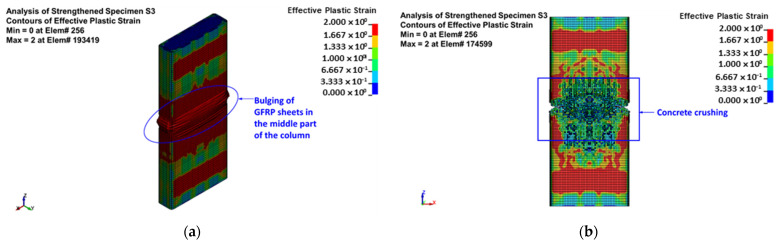

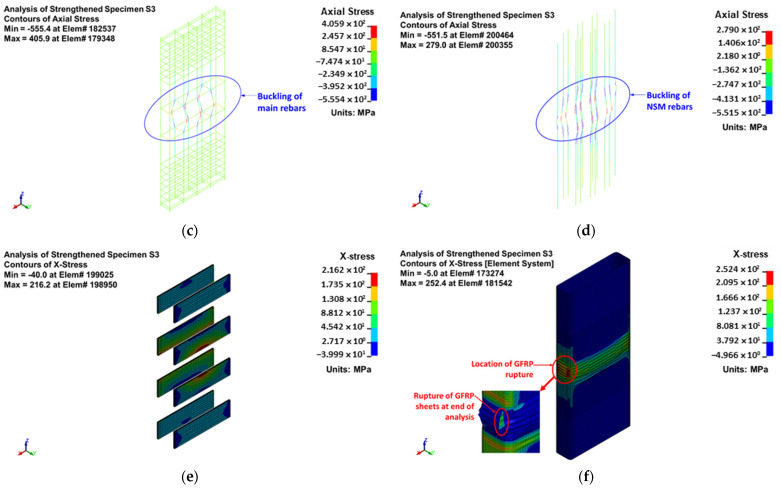
Numerically predicted failure mode for upgraded columns S3 and S4: (**a**) bulging of GFRP sheets at maximum load; (**b**) concrete damage contours; (**c**) stresses in reinforcement cage; (**d**) stresses in NSM bars; (**e**) stresses in steel plates; (**f**) stresses in the fiber direction for GFRP sheets.

The predicted failure modes are shown in Figure 15 for control column C and Figure 16, Figure 17 and Figure 18 for upgraded specimens S1 to S4, respectively. The FE failure modes are presented with regard to concrete damage contours (varying from 0 for the no-damage case to 2 for the full-damage case), contours of axial stress for steel and NSM bars, and contours of X-stress for steel plates and GFRP wrapping. It is identified that the predicted failure closely matched the observed failure of specimens seen earlier in Figure 10.

For control column C, the failure was predicted to occur owing to the buckling of vertical bars and crushing of concrete located in the central 600 mm length of the specimen. For retrofitted column S1, the failure was initiated by the bulging of the GFRP layers in the bottom region of the middle 600 mm length. The failure at the end of the analysis was because of the buckling of vertical bars and crushing of concrete, succeeded by the rupture of the GFRP sheets in the smaller side of the section, as presented in Figure 16.

For upgraded column S2, the failure started with the bulging of the GFRP sheets in the central region of the height between the bonded steel plates. The failure at the end of the analysis was because of the buckling of vertical bars and crushing of concrete, succeeded by the rupture of GFRP sheets close to the column corners, as seen in Figure 17.

As mentioned earlier, the numerical results of columns S3 and S3 are the same. Figure 18 depicts the numerically predicted failure mode for specimens S3 and S4. As seen from the figure, the failure started with the bulging of the GFRP sheets in the central region of the height between the bonded steel plates. At the end of the analysis, the failure was owing to the buckling of main and NSM steel bars and crushing of concrete, and it was then followed by rupture of the GFRP sheets at the column corners, as illustrated in Figure 18. This was confirmed by the FE maximum strain in the GFRP wrapping given in Table 4, since it was very close to the rupture value (=0.55 times the rupture strain of the flat GFRP coupons, as mentioned previously).

### 6.2. Load versus Displacement Curves

**Figure 19:**
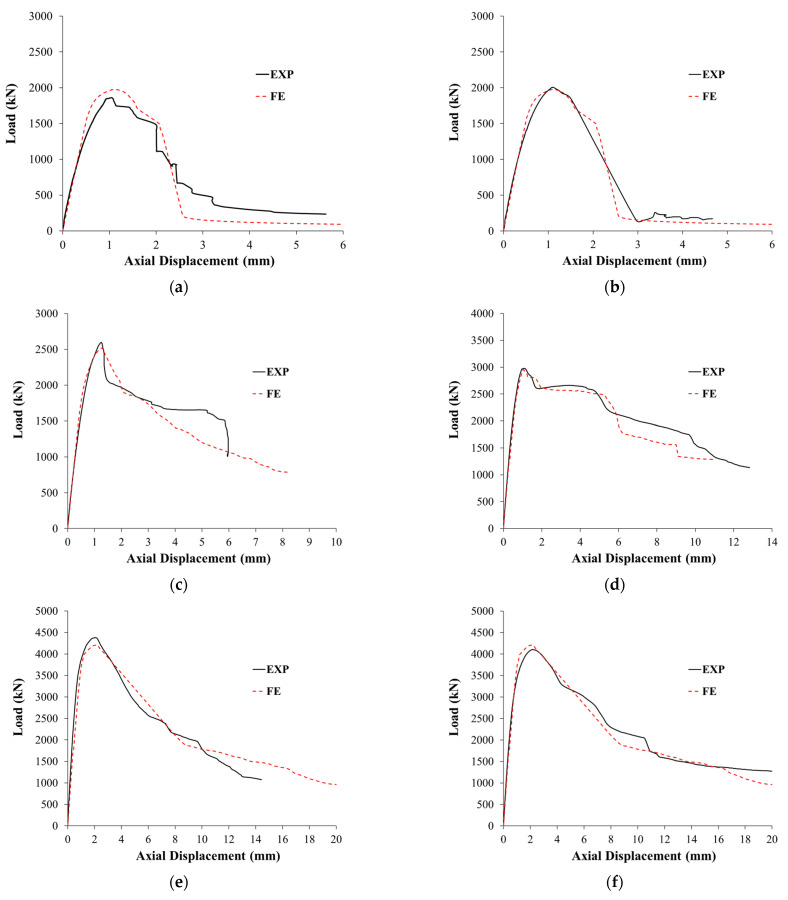
Experimental versus FE load–displacement plots for tested columns: (**a**) C1; (**b**) C2; (**c**) S1; (**d**) S2; (**e**) S3; (**f**) S4.

Figure 19 presents the comparisons between the numerical and experimental load versus axial displacement plots for tested specimens, and a good agreement was observed between the two plots for both control and upgraded specimens. The post-peak softening response in the experimental curves was also accurately predicted, which validates the employed models for constituent materials.

As noted from Figure 19c and Table 3, scheme 1 was numerically predicted to have a moderate improvement in the peak load, since the FE ultimate load of column S1 was 28% higher than that of the control column C. Figure 19d and Table 3 depict that scheme 2 was numerically predicted to be effective at improving the peak axial load of RC wall-like columns. The numerically predicted ultimate load of column S2 revealed improvements of about 49% and 17%, respectively, over the control specimen C and the upgraded column S1. It is also depicted in Table 3 and Figure 19d that the ultimate displacement of strengthened column S2 was considerably more than the control column C (by about 179%) and the strengthened column S1 (by about 100%). The addition of bolted steel plates significantly enhanced the axial displacement ductility of the wall-like column.

As depicted in Figure 19e,f, both schemes 3 and 4 (which utilized a hybrid system of NSM bars combined with GFRP wrapping and steel plates) were numerically predicted to significantly improve the load–displacement characteristics of the RC wall-like columns. The maximum predicted load of the strengthened columns S3 and S4 showed enhancements of about 113%, 67%, and 43% over the control column C, the upgraded specimen S1, and the strengthened column S2, respectively. It is also identified from Table 3 and Figure 19e,f that the use of bolted steel plates in schemes 3 and 4 was predicted to enhance the ultimate displacement considerably in comparison with the control column C (by about 191%) and the upgraded column S1 (by about 111%). However, the predicted ultimate displacements of specimens S3 and S4 were almost the same as the ultimate displacement of the upgraded specimen S2 (difference = 4%).

## 7. Evaluation of Upgrading Schemes

**Figure 20:**
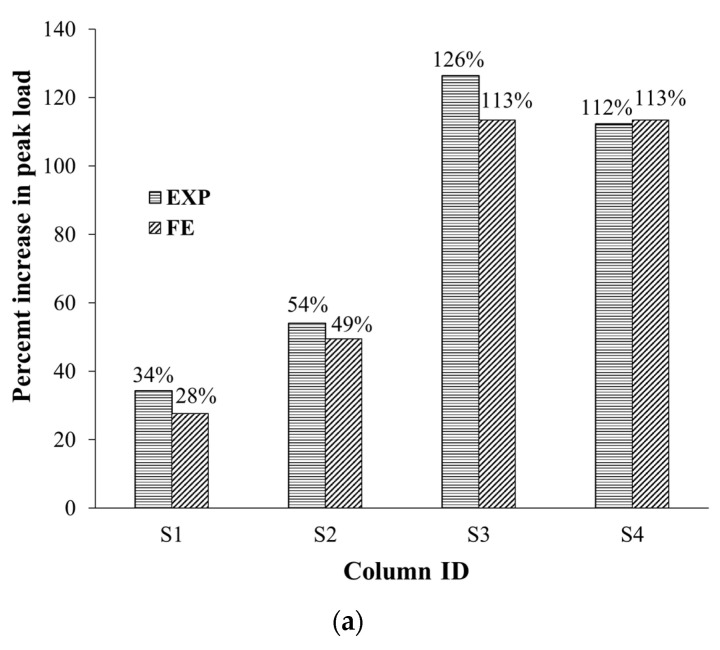

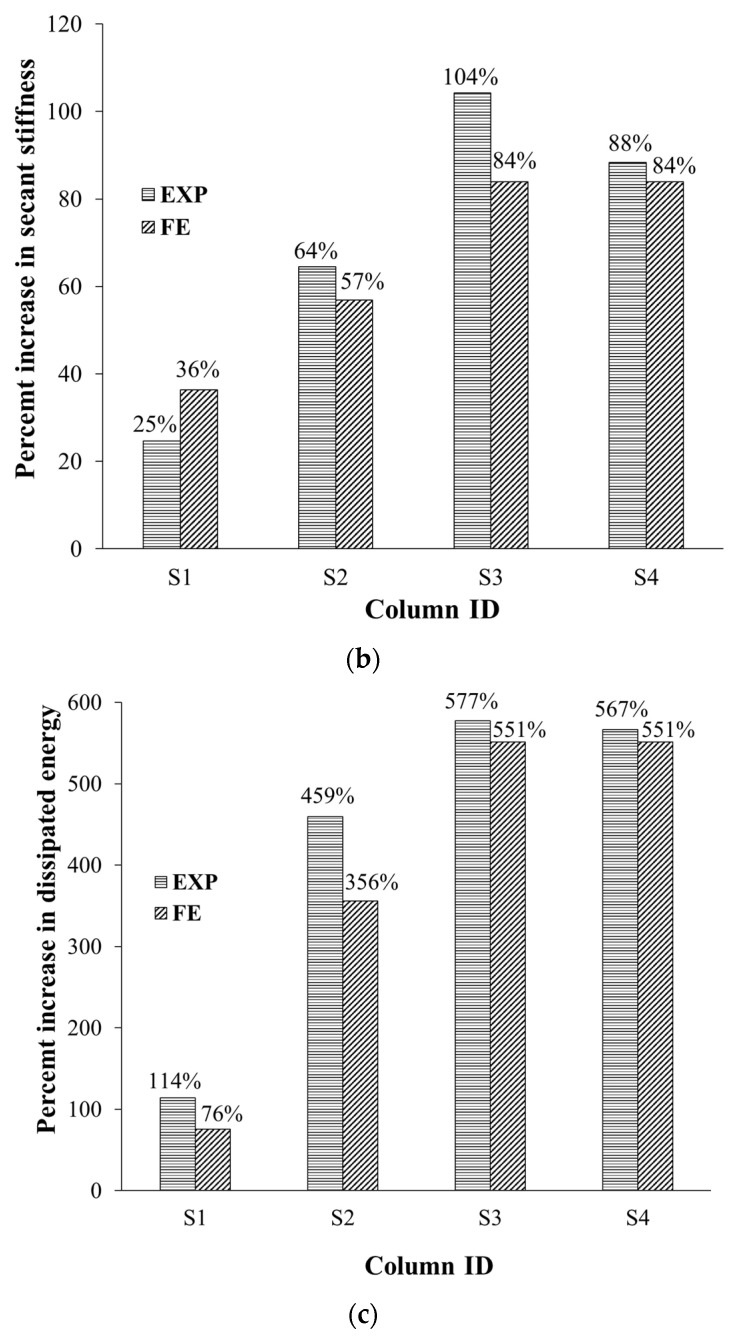
Comparison of upgraded columns with regard to the percent increase in (**a**) peak load, (**b**) axial stiffness, and (**c**) energy dissipated.

For comparison between the studied strengthening schemes 1 to 4, the bar charts in Figure 20 are plotted to show the impact of the upgrading scheme on the increase in peak load, axial stiffness, and dissipated energy with regard to the control columns. The bar charts are based on the experimental and FE results. It is evidenced that wrapping five GFRP layers around the middle portion in specimen S1 was moderately effective at enhancing the peak resistance by 34% and 28% for experimental and FE results, respectively. Nevertheless, combining bolted steel plates with GFRP wrapping in specimen S2 was effective at increasing the peak load by 54% and 49%, respectively, for test and numerical results. As seen in Figure 20a, the use of the hybrid system (NSM steel bars combined with GFRP wrapping and bolted steel plates) in specimens S3 and S4 was very effective at increasing the experimental peak load by 126% (113% for FE results) and 112% (113% for FE results), respectively. The best performance of schemes 3 and 4 could be owing to the enhanced concrete confinement provided by all of the GFRP composites, steel plates, and NSM bars, along with the increased axial load resisted by the NSM bars.

As shown in Figure 20b, wrapping five CFRP layers in specimen S1 had a small influence on improving the axial stiffness (the enhancements were 25% and 36% for test and numerical results, respectively). Nevertheless, combining bolted steel plates with GFRP wrapping in specimen S2 was effective at improving the axial stiffness by 64% and 57% for test and FE results, respectively, as illustrated in Figure 20b. The use of the hybrid system (NSM steel bars combined with GFRP wrapping and bolted steel plates) in specimens S3 and S4 was very effective at upgrading the axial stiffness by 104% for test results (84% for FE results) and 88% for test results (84% for FE results), respectively, as presented in Figure 20b. It is important to note that increasing the axial stiffness is preferred in multistory buildings as it will decrease the long-term creep deformation of RC columns. Figure 20c depicts the percent enhancement in the dissipated energy due to strengthening. As illustrated in the figure, very large enhancements in the dissipated energy were provided by schemes 2, 3, and 4 (varying from 507% to 577% and 356% to 551%, respectively, for test and numerical results); however, in scheme 1, the enhancements in the dissipated energy were 114% and 76% for test and numerical results, respectively.

**Figure 21:**
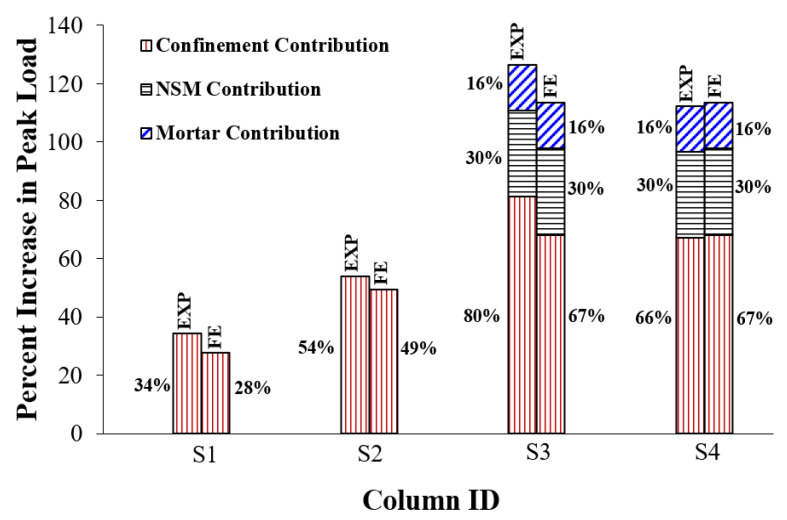
Effect of the contribution of different components on the percent increase in peak load for upgraded columns.

The effect of the contribution of different components on the percent increase in maximum load for each upgrading scheme (with regard to the control columns) is shown in Figure 21. The peak load enhancement in upgraded specimens is generally assumed to be divided into three components, which are enhancement provided by confinement of column section (for all schemes), enhancement provided by the axial resistance of NSM bars (for schemes 3 and 4), and enhancement provided by the increased mortar strength with respect to bare concrete of column section (for schemes 3 and 4). The share of each component was roughly computed from the following formulas:

(17) $$\%\;\mathrm{increase}\;\mathrm{due}\;\mathrm{to}\;\mathrm{upgrading}\;(\delta P)=\frac{\mathrm{Peak}\;\mathrm{load}\;\mathrm{of}\;\mathrm{upgraded}\;\mathrm{column}\;-\;\mathrm{Peak}\;\mathrm{load}\;\mathrm{of}\;\mathrm{control}\;\mathrm{column}}{\mathrm{Peak}\;\mathrm{load}\;\mathrm{of}\;\mathrm{control}\;\mathrm{column}}\times 100$$

(18) $$\%\;\mathrm{increase}\;\mathrm{due}\;\mathrm{to}\;\mathrm{NSM}\;\mathrm{bars}\;(\delta P_{NSM})=\frac{A_{NSM}f_{y-NSM}}{\mathrm{Peak}\;\mathrm{load}\;\mathrm{of}\;\mathrm{control}\;\mathrm{column}}\times 100$$

(19) $$\%\;\mathrm{increase}\;\mathrm{due}\;\mathrm{to}\;\mathrm{mortar}\;(\delta P_{mortar})=\frac{0.85\left(f_{m}^{\prime }-f_{c}^{\prime }\right)\left(A_{grooves}-A_{NSM}\right)}{\mathrm{Peak}\;\mathrm{load}\;\mathrm{of}\;\mathrm{control}\;\mathrm{column}}\times 100$$

(20) $$\%\;\mathrm{increase}\;\mathrm{due}\;\mathrm{to}\;\mathrm{confinement}\;(\delta P_{conf})=\delta P-\delta P_{NSM}-\delta P_{mortar}$$

where *A_grooves_* is the area of grooves for NSM bars and $f_{m}^{\prime }$ is the mortar compressive strength.

It is clarified from Figure 21 that the addition of bolted steel plates in column S2 enhanced the section confinement with the peak load enhancements (compared with control columns) increased from 34% (for specimen S1) to 54% and 28% (for specimen S1) to 49% for test and numerical results, respectively. The addition of NSM bars in specimens S3 and S4 added more confinement to the column section by increasing the experimental peak load enhancement from 54% (for specimen S2) to 80% and 66%, respectively. For both specimens S3 and S4, it was also predicted that the addition of NSM bars provided more section confinement and the confinement contribution increased from 49% (for specimen S2) to 67%. As clarified earlier from Figure 20a, schemes 3 and 4 had experimental peak load enhancements of 126% and 112%, respectively. As presented in Figure 21, these enhancement ratios are divided into 80% and 66%, respectively, provided by section confinement, 30% contributed by the axial resistance of NSM bars, and the remaining 16% provided by the increased strength of mortar. However, for both schemes 3 and 4 a maximum load increase of 113% was predicted, which is divided into 67% added by section confinement, 30% provided by NSM bars, and 16% added by the enhanced mortar strength.

## 8. Conclusions

In this research, a total of six RC wall-like columns were studied experimentally under concentric compression. Two specimens were un-retrofitted to be used as control columns. The remaining four specimens were retrofitted with four schemes. The schemes incorporated wrapping GFRP sheets around the column versus hybrid steel/NSM/GFRP systems. Besides the testing program, two analytical approaches were suggested for evaluating the axial capacity of tested columns. Moreover, 3D nonlinear FE analysis was performed for assessing the axial load versus displacement behavior of tested specimens. The main outcomes of this research are as follows:

1. The strengthening schemes proposed in this research for RC wall-like columns were designed so that the dimensions of the column cross-section are not increased. Accordingly, they are favored by architects, especially in buildings constructed in congested and expensive areas of metropolitan cities.
2. Control specimens had a common brittle failure mode that started with cover spalling, resulting in buckling of vertical bars, and the failure was concluded by crushing concrete in the middle part of the height. For all strengthened columns, the failure started with the bulging of the GFRP sheets in the middle part of the column height, and it was followed by the buckling of both NSM bars (if any) and main steel bars. Then, the failure was concluded by rupture of GFRP wrapping at (or close to) the corners.
3. Upgrading of wall-like columns by GFRP wrapping in scheme 1 was moderately efficient in increasing the maximum load by 34% and 28%, respectively, for test and numerical results. It also had a small influence on increasing the axial column stiffness (the enhancements were 25% and 36% for test and numerical results, respectively).
4. Combining bolted steel plates with GFRP wrapping in scheme 2 was effective at improving the ultimate load by 54% and 49% for test and numerical results, respectively. It was also effective at increasing the axial stiffness of the column by 64% and 57% for test and FE results, respectively.
5. Among the four studied schemes, the use of the hybrid system (GFRP wrapping along with bolted steel plates and NSM bars) in schemes 3 and 4 was the most effective at increasing the maximum experimental load by 126% (113% for FE results) and 112% (113% for FE results), respectively. The best performance of schemes 3 and 4 could be owing to the enhanced concrete confinement provided by all of the GFRP composites, steel plates, and NSM bars, along with the increased axial load resisted by the NSM bars. In addition, the use of the hybrid system in specimens S3 and S4 was very effective at upgrading the experimental axial stiffness by 104% (84% for FE results) and 88% (84% for FE results), respectively.
6. Schemes 2, 3, and 4, which involved the use of bolted steel plates combined with GFRP wrapping, had substantial enhancement in the ultimate axial displacement of the columns. Compared with control columns, ultimate displacement enhancements of 157% to 253% and 179% to 191% were provided by schemes 2, 3, and 4 for experimental and FE results, respectively.
7. Two analytical approaches were developed for computing the ultimate load of control and upgraded RC wall-like columns. Then, the calculated loads were compared with the test values. The first analytical approach predicted the maximum load of control columns more precisely than the second one. Nevertheless, Approach 2 predicted the maximum load of upgraded columns more accurately than Approach 1. For upgraded columns, the prediction errors of Approach 1 were 20% to 35%; nevertheless, the errors of Approach 2 were 3% to 14%.
8. The FE results with regard to failure modes and load–displacement behavior accurately matched the test results. This validates the employed models of constitutive materials (concrete, GFRP sheets, and steel). Thus, the conducted FE analysis can be utilized in forthcoming studies on the strengthening of RC columns.

## Figures and Tables

**Table 2:** Key FE input parameters for constituent materials.

**Concrete and Mortar**	**Concrete**		**Epoxy-Based Mortar**	
Model No.	72R3			
Model type	Concrete damage			
Compressive strength	29.2 MPa		65.0 MPa	
Aggregate size	10.0 mm		5.0 mm	
**Steel reinforcing bars, rods, and plates**	**Φ8 mm bars**	**Φ10 mm bars**	**Φ18 mm rods**	**Plates**
Model No.	24			
Model type	Piecewise linear plasticity			
Modulus of elasticity	2 × 10^5^ MPa			
Yield strength	548.0 MPa	531.0 MPa	711.0 MPa	230.0 MPa
Tangent modulus	86.4 MPa	133.8 MPa	0.0	0.0
Plastic fracture strain	9.72%	9.73%	6.64%	19.89%
**GFRP sheets**				
Model No.	54–55			
Model type	Enhanced composite damage			
Density	1740 kg/m^3^			
Thickness per layer	1.30 mm			
Elastic modulus in the fiber direction	20.90 × 10^3^ MPa			
Elastic modulus in the transverse direction	1.05 × 10^3^ MPa			
Tensile strength in the fiber direction	253.0 MPa			
Tensile strength in the transverse direction	25.30 MPa			

**Table 3:** Key results of load versus displacement characteristics for tested specimens.

Specimen ID	Results	Yield Load (kN)	Peak Load (kN)	Axial Displacement (mm) at				Axial Stiffness (kN/mm)	Dissipated Energy (kN.mm)
				Service Load	Yield Load	Peak Load	Ultimate State		
Control specimens									
C1	TEST	1846	1862	0.27	0.93	1.05	2.01	2810	2866
	FEM	1969	1974	0.29	1.04	1.09	2.15	2693	3220
	*TEST/FEM*	*0.94*	*0.94*	*0.90*	*0.89*	*0.96*	*0.93*	*1.04*	*0.89*
C2	TEST	1919	2006	0.29	0.93	1.11	1.97	2815	3157
	FEM	1969	1974	0.29	1.04	1.09	2.15	2693	3220
	*TEST/FEM*	*0.97*	*1.02*	*0.97*	*0.89*	*1.02*	*0.92*	*1.05*	*0.98*
Mean of C1 and C2	TEST	1883	1934	0.24	0.93	1.08	1.99	3309	3012
	FEM	1969	1974	0.25	1.04	1.09	2.15	3154	3220
	*TEST/FEM*	*0.96*	*0.98*	*0.94*	*0.89*	*0.99*	*0.93*	*1.05*	*0.94*
Strengthened specimens									
S1	TEST	2470	2596	0.30	1.06	1.25	3.5	3505	6450
	FEM	2391	2519	0.27	0.98	1.29	2.96	3673	5663
	*TEST/FEM*	*1.03*	*1.03*	*1.08*	*1.08*	*0.97*	*1.17*	*0.95*	*1.14*
S2	TEST	2637	2978	0.26	0.73	1.11	7.02	4626	16,845
	FEM	2807	2950	0.28	0.89	1.02	6.01	4225	14,678
	*TEST/FEM*	*0.94*	*1.01*	*0.92*	*0.82*	*1.08*	*1.17*	*1.09*	*1.15*
S3	TEST	3647	4379	0.31	0.84	2.06	5.11	5743	20,396
	FEM	3753	4212	0.34	1.03	2.20	6.25	4954	20,967
	*TEST/FEM*	*0.97*	*1.04*	*0.90*	*0.82*	*0.94*	*0.82*	*1.16*	*0.97*
S4	TEST	3350	4105	0.31	0.94	2.20	6.00	5297	20,073
	FEM	3753	4212	0.34	1.03	2.20	6.25	4954	20,967
	*TEST/FEM*	*0.89*	*0.97*	*0.91*	*0.92*	*1.00*	*0.96*	*1.07*	*0.96*

**Table 4:** Key results of stress versus strain response for tested specimens.

Specimen ID	Results	Average Peak Stress (MPa)	Actual Peak Stress (MPa)	Concrete Strain at Peak Stress (με)	Ultimate Concrete Strain (με)	Strain in Main Bars at Peak Load (με)	Strain in NSM Bars at Peak Load (με)	Peak GFRP Strain (με)	Peak Steel Plate Strain (με)
Control specimens									
C1	TEST	29.79	23.41	2600	5000	3400	-	-	-
	FEM	31.59	25.23	2700	5400	3300	-	-	-
	*TEST/FEM*	*0.94*	*0.93*	*0.96*	*0.93*	*1.04*	*-*	*-*	*-*
C2	TEST	32.09	25.74	2800	4900	3100	-	-	-
	FEM	31.59	25.23	2700	5400	3300	-	-	-
	*TEST/FEM*	*1.02*	*1.02*	*1.02*	*0.92*	*0.95*	*-*	*-*	*-*
Mean of C1 and C2	TEST	30.94	24.58	2700	4950	3250			
	FEM	31.59	25.23	2700	5400	3300			
	*TEST/FEM*	*0.98*	*0.97*	*1.00*	*0.92*	*0.98*			
Strengthened specimens									
S1	TEST	41.77	35.51	3128	8678	3195	-	10,274	-
	FEM	40.30	34.25	3230	7392	3579	-	12,088	-
	*TEST/FEM*	*1.04*	*1.04*	*0.97*	*1.17*	*0.89*	*-*	*0.85*	*-*
S2	TEST	47.91	41.73	2763	17,544	6205	-	12,451	4033
	FEM	47.21	41.28	2550	15,016	6648	-	12,083	3558
	*TEST/FEM*	*1.01*	*1.01*	*1.08*	*1.17*	*0.93*	*-*	*1.03*	*1.13*
S3	TEST	70.45	56.05	5147	12,763	11,357	12,376	14,369	3432
	FEM	67.39	53.29	5488	15,622	12,269	13,669	12,075	3845
	*TEST/FEM*	*1.05*	*1.05*	*0.94*	*0.82*	*0.93*	*0.91*	*1.19*	*0.89*
S4	TEST	66.04	51.51	5509	15,003	13,491	13,184	11,543	3225
	FEM	67.39	53.29	5488	15,622	12,269	13,669	12,075	3845
	*TEST/FEM*	*0.98*	*0.97*	*1.00*	*0.96*	*1.10*	*0.96*	*0.96*	*0.84*

**Table 5:** Calculation of ultimate load of tested specimens by analytical models.

Specimen ID	Approach 1		Approach 2	
	*P_u-ana_* (kN)	*P_u-exp_/P_u-ana_*	*P_u-ana_* (kN)	*P_u-exp_/P_u-ana_*
C1	1946	0.96	2216	0.84
C2	1946	1.03	2216	0.91
Mean of C1 and C2	1946	0.99	2216	0.87
S1	1983	1.31	2285	1.14
S2	2198	1.35	2902	1.03
S3	3413	1.28	3927	1.11
S4	3413	1.20	3927	1.05

*P_u-exp_* = ultimate experimental load, *P_u-ana_* = ultimate analytical load.

## Data Availability

Not applicable.

## List of Nomenclature

*A_g_*	gross area of column section
*A_grooves_*	area of grooves for NSM bars
*A_NSM_*	area NSM bars
*A_st_*	area of parent steel bars
*b_p_*	width of steel plates
*B*	width of column cross-section
*D_e_*	diagonal of the rectangular column cross-section
*E_f_*	modulus of elasticity of GFRP sheets
*E_s_*	Young’s modulus of steel plates
$f_{c}^{\prime }$	unconfined compressive strength of concrete
$f_{c-act}^{\prime }$	maximum actual concrete strength
$f_{c-avg}^{\prime }$	maximum average concrete strength
$f_{cc}^{\prime }$	confined compressive strength of concrete
$f_{cc-grout}^{\prime }$	confined grout mortar strength (used in the NSM grooves)
$f_{𝓁}$	peak lateral confining pressure exerted on concrete
$f_{m}^{\prime }$	compressive strength of mortar
*f_y-NSM_*	yield strength of NSM bars
*f_y-st_*	yield strength of parent steel bars
*H*	depth of column cross-section
*k_a_*	FRP confinement efficiency factor
*k_s_*	axial secant stiffness
*P_s_*	service load
*P_u_*	peak load
*P_u-exp_*	ultimate experimental load
*P_u-ana_*	ultimate analytical load
*S_p_*	spacing of steel plates measured on centers
*r_c_*	radius of corner of column cross-section
*t_f_*	total thickness of GFRP layers
*t_p_*	thickness of steel plates
*P*	increase in load carrying capacity of column due to upgrading
*P_NSM_*	increase in load carrying capacity of column due to NSM bars
*P_mortar_*	increase in load carrying capacity of column due to mortar
*P_conf_*	increase in load carrying capacity of column due to confinement
*η*	equivalent rectangular stress block parameter
Ψ*_f_*	strength reduction factor for FRP confinement (=0.95)
*Δ_s_*	axial displacement at service load
*ε_fe_*	effective strain of GFRP sheets (=55% of the rupture strain)
*ρ_g_*	reinforcement ratio of longitudinal steel bars = *A_st_/BH*
